# Nitrogen removal from wastewater through microbial electrolysis cells and cation exchange membrane

**DOI:** 10.1186/2052-336X-12-48

**Published:** 2014-02-17

**Authors:** Sakineh Haddadi, GholamReza Nabi-Bidhendi, Nasser Mehrdadi

**Affiliations:** 1 Department of Environmental Engineering, Faculty of Environment, University of Tehran, Tehran, Iran

**Keywords:** Microbial electrolysis cell, Ammonium, Urine, Diffusion, Closed-circuit potential, Open-circuit potential

## Abstract

Vulnerability of water resources to nutrients led to progressively stricter standards for wastewater effluents. Modification of the conventional procedures to meet the new standards is inevitable. New technologies should give a priority to nitrogen removal. In this paper, ammonium chloride and urine as nitrogen sources were used to investigate the capacity of a microbial electrolysis cell (MEC) configured by cation exchange membrane (CEM) for electrochemical removal of nitrogen over open-and closed-circuit potentials (OCP and CCP) during biodegradation of organic matter. Results obtained from this study indicated that CEM was permeable to both organic and ammonium nitrogen over OCP. Power substantially mediated ammonium migration from anodic wastewater to the cathode, as well. With a urine rich wastewater in the anode, the maximum rate of ammonium intake into the cathode varied from 34.2 to 40.6 mg/L.h over CCP compared to 10.5-14.9 mg/L.h over OCP. Ammonium separation over CCP was directly related to current. For 1.46-2.12 mmol electron produced, 20.5-29.7 mg-N ammonium was removed. Current also increased cathodic pH up to 12, a desirable pH for changing ammonium ion to ammonia gas. Results emphasized the potential for MEC in control of ammonium through ammonium separation and ammonia volatilization provided that membrane characteristic is considered in their development.

## Introduction

Wastewater treatment technologies are energy demanding processes. As an example, 3-4% of the total energy demand in the United Kingdom comes from wastewater processes. Also, the Unites States consumes 21 billion kwh for this purpose [1]. Nitrogen removal in wastewater treatment is a main concern. The majority of nitrogen in municipal wastewater originates from urine. An adult person typically produce an average of 11.5 g-N/d urine [2]. Nitrogen release into environment could lead to autrification in water reservoirs. Most treatment methods have mainly been established for organic removal. Recent standards have forced treatment processes to be enhanced for nitrogen elimination. On the other hand, extra considerations should be taken into account when nutrient removal is desirable beside organic removal; this complicates the treatment method [3] and poses extra costs. Hence, urine separation at the source can hold the promise of sustainability of wastewater management [4].

Bioelectrochemical systems convert organic matter to protons and recoverable electrons within an anode compartment. Electrons flow through an external circuit to cathode and conduct reduction reactions. Usually hydroxide ions are produced from oxygen reduction in the cathode [5]. Simply, two configurations of BESs are used for wastewater treatment purposes: microbial fuel cell (MFC) or microbial electrolysis cell (MEC). Anodic reactions are almost the same in both configurations. Anode and cathode are connected to each other by a load in MFC; whereas, in MEC, anode potential adjusted to a specific amount or external voltage is invested to prepare enough energy for accomplishment of reactions in the cathode to produce hydrogen gas [6]. These systems have been tested for purification of synthetic and real wastewaters, organic and inorganic pollutants [7,8]. They were able to capture energy from treatment of domestic wastewater [9], food-processing wastewater [10], landfill leachate [11], cellulose [12], and even alcohol [13,14]. They have shown recycling energy from waste and it is time to evaluate their capability for removal of pollutants that might not have any energy content but are also environmentally important such as nitrogen compounds.

Once removing nitrogen from wastewater using bioelectrochemical systems comes to mind, the bioremediation aspect of BESs might be highlighted. During bioremediation, nitrate is used in the biocathode, acts as the final electron acceptor and is reduced to nitrite or nitrogen gas [15,16]. In fact, bacteria take electrons from electrodes and participate in nitrate reduction. Biological standard potential (at pH = 7) of NO_3_^-^/NO_2_^-^ and NO_3_^-^/N_2_ are +0.43 V and +0.74 V respectively; while, this potential for the commonly oxidant in the MFC, O_2_/H_2_O, is +0.82 V [17,18]. In BES systems anode compartment is operated in anaerobic condition [19]. Oxygen or oxygen compounds interfere with energy production process [20]. Consequently, biological removal of nitrate is not suggested in the bioanode. Moreover, lack of oxygen in the anode prevents nitrification. In theory, bioanode seems to have the least chance for dealing with this nutrient since it makes nitrification or denitrification impossible. However, oxygen leakage from air-cathode might contribute to nitrification [21]. Nitrification and denitrification is the widely used concept for nitrogen removal in biological technologies. However, it should be noted that this process has some limitations for nitrogen rich wastewaters. Physicochemical processes also can remove nitrogen from wastewater but they are much more expensive than biological methods. Bioelectrocemical systems as their name implies are not sole biological methods. They also involve physical and chemical reactions to clean wastewater and produce energy.

Beside electrons, organic biodegradation in BES is also associated with proton production. As electrons leave the anode, charge would be imbalanced in the system. Then protons might also immigrate to the cathode; otherwise, some negative charges from the cathode should come to the anode to account for charge neutrality. In most designs, a membrane separates anode and cathode cells and so charges must cross the permeable membrane. Hydroxyl ions or protons are responsible for this charge neutrality, but other cations or anions may also be involved. Some studies have reported ions traveling other than protons or hydroxide [22]. Hence, the use of membrane in BES is sometimes controversial [23].

Despite the fact that bioelectrochemical systems have potential for removing organic matters and producing energy, simultaneous biotransformation of organic waste and nutrient from wastewater in one bio-cell seems to be impractical. In addition, nutrient removal and scaling up remain unaddressed. Nevertheless, bio-mediated electrochemical removal of nitrogen in the bioanode, if feasible, can overcome those limitations and enables simultaneous nitrogen and organic matter removal in the anode. Ion transfer instead of charge transfer can provide a chance for taking off some unpleasant ions from wastewater. Here, in this study, MEC system configured by cation exchange membrane (CEM) was tested to remove nitrogen as ammonium ion from ammonium and urine-rich wastewaters.

Cation exchange membranes are supposed to exchange ions but this study would have a look at its permeability to organic nitrogen when faced with urine, a rich source of organic nitrogen.

## Material and methods

### Media

The main sources of nitrogen in urine are urea and creatinine. Their concentration for producing mimic urine were chosen based on the literature [24], 25 g/L urea and 1.1 g/L creatinine. Except mimic urine, two other media were also used (Table 1). These three media were used to study: 1- Nitrogen transport though membrane because of gradient concentration in a representative mimic urine. 2- Nitrogen transport through membrane in urine rich wastewater where diffusion is combined by current generation 3- Nitrogen transport through membrane in an ammonium rich wastewater (containing ammonium chloride), in which ammonium content is almost stable in the wastewater and does not change because of biological activity under anaerobic conditions. Two different acetate concentrations, 20 and 25 mM, were used to create different current output. For each purpose, most experiments were performed in duplicates. Medium pH of ammonium rich wastewater was adjusted to ~ 7 with NaOH when needed.

**Table 1:** Medium compositions

**Compound**	**Mimic urine**	**Urine and acetate**	**Concentration g/L Ammonium and acetate (20 mM)**	**Ammonium and acetate (25 mM)**
KH_2_PO_4_	3.00	3.00	1.82	2.27
Na_2_HPO_4_-12H_2_O	15.4	2.67	9.34	11.64
NH_4_CL	0.049	0.017	11.5	11.5
MgCl_2_-6H_2_O	0.033	0.011	0.02	0.037
Urea	25.0	4.375	-	-
Creatinine	1.10	0.19	-	-
Acetate	-	0.54	1.64	2.05
Mineral solution	1 mL	0.5 mL	1 mL	1.25 mL
pH	7.0	6.6	7.5	7.5

### MEC configuration and operation

**Figure 1:**
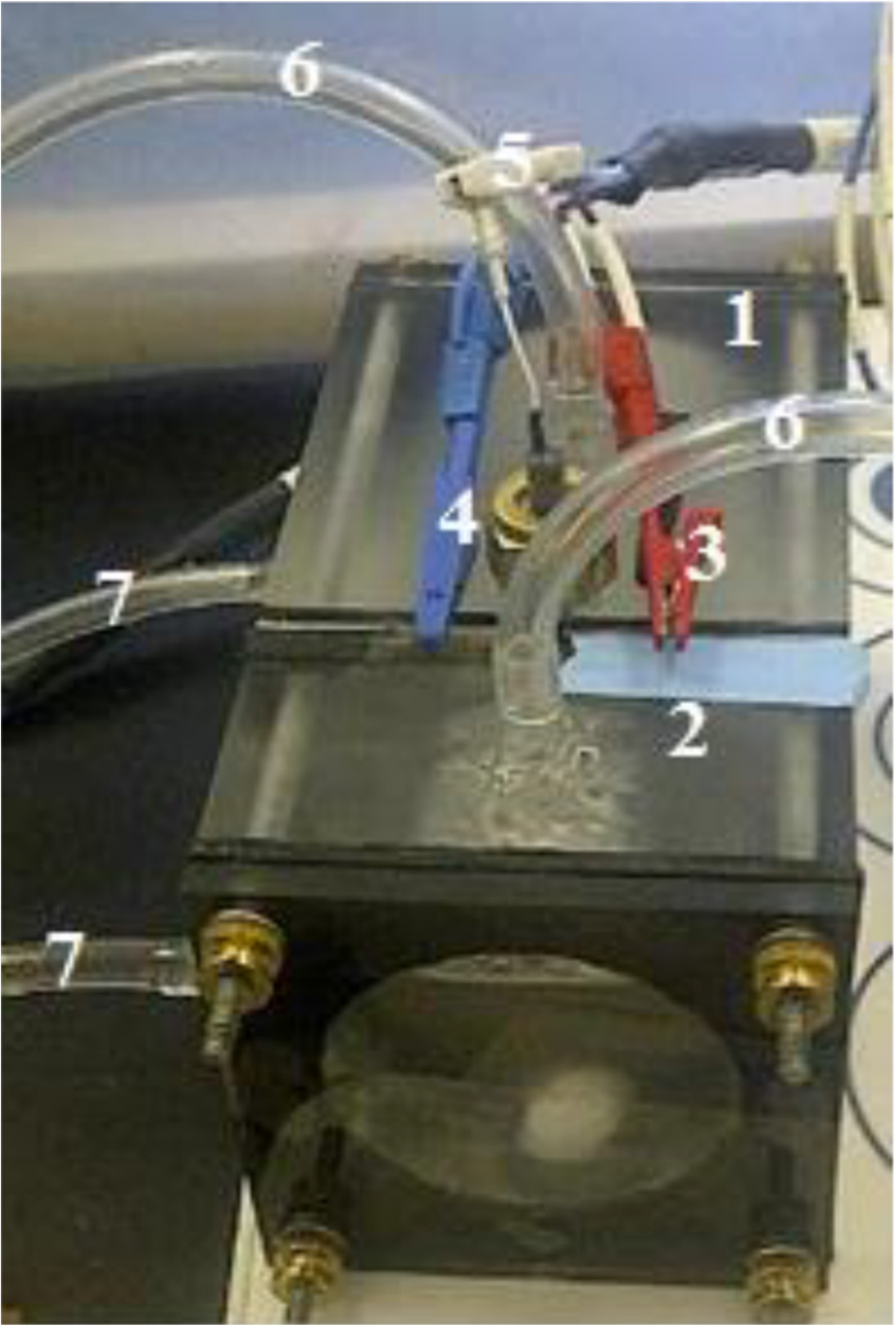
Reactor configuration from the top view: 1) anode chamber, 2) cathode chamber, 3) anode electrode connected to potentiostat, 4) cathode electrode connected to potentiostat, 5) reference electrode, 6) outlet tubes, 7) inlet tubes.

Microbial electrolysis cells consisted of two-chamber, cylindrical reactors with a membrane between compartments (Figure 1). For urea and ammonia tests, reactors were different in membrane area and a little in dimensions but similar in shape and electrode positions. In one of them, anode chamber of 10.24 cm was connected to 4.52 cm cathode chamber with contact area (membrane area) of 32.1 cm^2^; while, in the other one, anode and cathode lengths and contact area were 10.62 and 4.42 cm and 28.26 cm^2^, respectively. Both reactors had working volumes of 290 for anode and 120 mL for cathode. Cation exchange membrane (CEM) was located between chambers and was sealed with rubber and Vaseline as gaskets. Rubber bands were also placed between chambers and electrodes. A stainless steel plate with the same open area as the membrane surface area and integrated with carbon fibers worked as anode. Right after the gasket a stainless steel mesh was another electrode (cathode). An AgCl reference electrode was placed in 1 cm distance of anode. Using a potentiostat, anode potential was set at -0.4 mV versus this reference electrode under closed circuit mode. The three-channel potentiostat hooked up to a personal computer recorded current continuously every two minutes. These reactors were also operated in opened-circuit potential (OCP), without connecting to the potentiostat or putting an external load between anode and cathode. The operations were done at 25 ±1°C and in batch mode. Anode was inoculated with MEC effluent and anaerobic sludge. To trace nitrogen in the cathode, cathode was filled with deionized water. Both chambers were operated in anaerobic condition. Blowing nitrogen gas for an hour at the beginning of each run emitted oxygen from solutions and ensured anaerobic condition.

### Measurements and calculations

Current and cumulative current directly were recorded by potentiostat. The power densities (mW/cm^2^) were calculated as $P=\frac{\mathrm{IE}}{A}$, where A is surface area of membrane (cm^2^), I is current (mA), E is voltage (V). To equalize energy production and nitrogen removal unites, total current produced in a given time was also presented as mmol electron (e) as following:

(1) $$e=\frac{\mathrm{dQ}}{F}$$

dQ states cumulative current (mA.s) and F is Farady’s constant (96485 A.s/mol).

pH was measured by a pH meter. For urine experiments, ammonium and TKN were traced every day almost over a one-week period. Ammonium in ammonium chloride fed reactors was traced over 24 hours with 4 hour-intervals.

Total Kjeldahl Nitrogen (TKN) and ammonium nitrogen (NH4-N) were determined via colorimetry method by an ammonia analyzer (Bran + Luebbe AutoAnalyzer 3). Prior to quantifying TKN, samples were digested at 200°C for 1.5 hours and at 350°C for 3.5 hours in sulfuric acid and left overnight to be cooled. Organic nitrogen was calculated as:

(2) $$N_{\mathrm{org}}=\mathrm{TKN}‒N_{NH_{4}}$$

mmol NH_4_ transferred at time t was estimated by:

(3) $$W=\frac{\sum_{i}^{t}C_{t}V_{t}}{M_{W}}$$

Where, parameters are: W ammonium mass (mmol), C_t_ ammonium concentration at time t (mg-N/L), V_t_ water volume in the cathode at time t (L), M_w_ molecular weight of nitrogen (14 g/mol).

## Results and discussion

### Nitrogen removal from mimic urine medium by CEM

**Figure 2:**
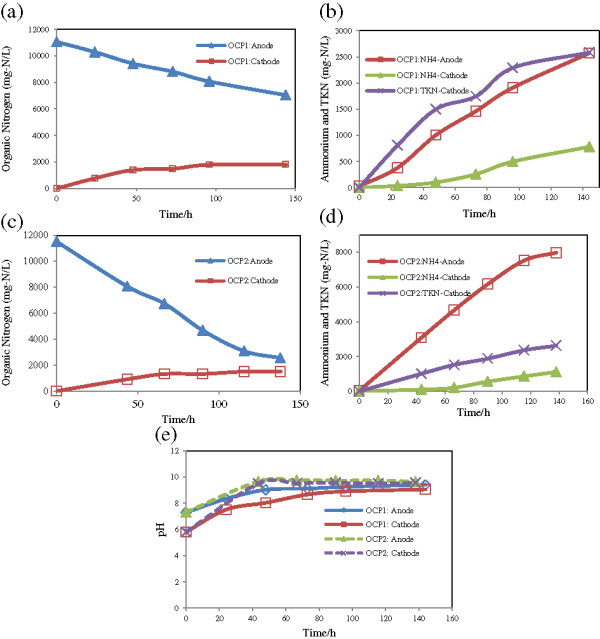
Changes of nitrogen species and pH in two runs operated in open-circuit potential, using urine medium (11 g-N/L): a & c) organic nitrogen, b & d) TKN and ammonium, e) pH.

Synthetic urine containing 11 g-N/L was used to follow nitrogen removal across CEM membrane. At the beginning, all of TKN in the anode was as organic form. Organic nitrogen (N_org_) gradually moved to the cathode because of concentration gradient. This caused catholyte to have 1400 mg-N/L after 48 hours of operation (Figure 2a). Urine in a bioreactor is usually unstable and readily decomposed into NH_4_-N. Anolyte urine started being hydrolyzed but there was still 80% unchanged after 100 h of operation. At this time, N_org_ of catholyte just had increased 400 mg/L more rather than 48 hour. There was no more increase afterwards.

Following appearance of ammonium in the anode, it was transferred to the cathode. Figure 2b shows catholyte ammonium as a function of time and anolyte ammonium. At the end of operation, ammonium in the anode was found to be 2580 mg-N/L that resulted in a 782 mg-N/L cumulative ammonium in the cathode.

The test was replicated to ensure the repeatability of the test results. Some differences were observed between two series runs with similar urine concentration owing to urine dissociation rate. In the second run, however, urine decomposition took place faster which might be related to bacteria activity. More than 30% of the TKN was as ammonium after 45 h of operation and it approached 80% over 62 h. Such as previous run, most of the organic nitrogen was transferred to the cathode within the first two days. The maximum organic nitrogen in the cathode was 1500 mg/L and took place over 138 h (Figure 2c); parallelly, NH_4_-N was also migrated and reached to 1120 mg/L (Figure 2d).

Reactors fed by mimic urine showed that nitrogen in organic and ammonium forms could cross cation exchange membrane. Ammonium transfer is consistent with the characteristic of this type of membrane; but organic nitrogen is not supposed to be able to go through the membrane. Over 100 hours, the cathode TKN concentration was around 20% of the anode TKN. However, it does not mean that 20% of the TKN of anolyte went to the cathode, as the cathode was smaller than the anode. In both runs, organic nitrogen was transferred to the cathode exceeded ammonium nitrogen. The reason behind this might be its concentration. The driving force for nitrogen transfer was concentration gradient. The highest gradient between two chambers was at the beginning or in the first two days of the operation over which organic nitrogen was the prominent species. It constitutes 70-90% of the TKN during the first two days. As nitrogen left the anode toward the cathode, gradient concentration decreased and slowed further nitrogen transfer rate.

In addition to TKN, water could also pass through the membrane. Water, as expected in osmosis phenomenon, moved in the opposite direction of ions through the membrane. As the cathode was filled with deionized water, high gradient concentration was created between catholyte and anolyte. Then, the anode pulled water from the cathode. Nitrogen transfer might not have been significant, but when water left the cathode, cathodic ammonium seemed more concentrated than it really was. Unfortunately, we did not measure water transfer rate, but in two other experiments running with 11 g-N/L we had to add 15 mL water to the cathode, after 5 days of operation, to compensate water loss. Water loss would mislead in showing concentration based on *C*_1_*V*_1_ = *C*_2_*V*_2_ relationship in which parameters are: C_1_ real concentration, V_1_ initial volume, C_2_ the measured concentration, and V_2_ remained volume after water loss or intake.

Water and even acetate passed through the nafion 117 membrane [25,26], a proton exchange membrane which typically is used in BES studies. Rozendal et al. (2007) observed 33.5 ml water loss through nafion membrane having surface area of 256 cm^2^ over 48 h.

Ammonium content or type of nitrogen did not affect TKN transfer as can be driven from Figures (2b and 2d) starting with the same urine concentration. After 100 hours of operation, anodic ammonium increased to 2000 mg-N/L and 6500 mg-N/L while catholyte TKN was similar and respectively 2289 and 2363 mg/L.

**Table 2:** Maximum rates of ammonium increase in the cathode compartment

**Maximum rates (mg/L.h)**	**OCP1**	**OCP2**	**CCP1**	**CCP2**
Over 24 h	10.47	14.93	40.6	34.25
Over 48 h	8.25	13.29	27.5	26.90

Following the urine dissociation, pH of the solutions changed. Figure 2e shows pH changes of anolyte due to ammonium production. pH increased up to 9.7 and 9.5 in the anode and cathode, respectively.

Current was unattainable with this urine medium under the stated operation condition. The medium composition to attain current was also changed. To trace nitrogen over current generation process, some pretests were conducted and they revealed that MEC was able to produce current from a medium containing 4000 mg-N/L urine and acetate as an electron donor.

### Nitrogen transfer during closed-circuit voltage from urine rich substrate

**Figure 3:**
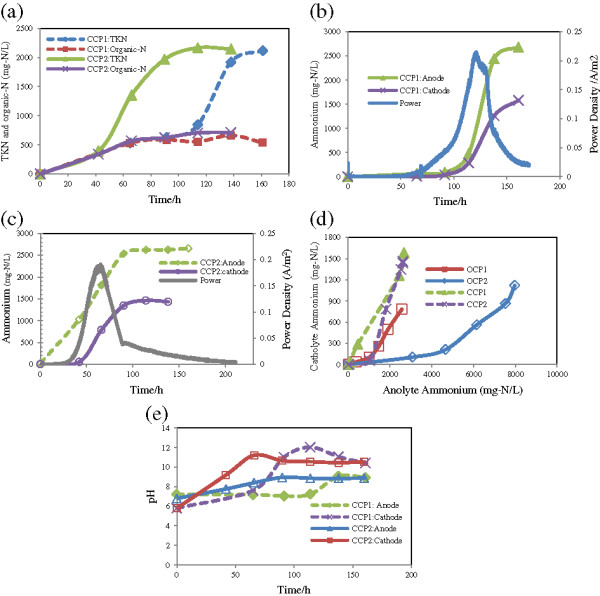
Variables over closed-circuit potential using urine and acetate medium: a) nitrogen species, b & c) power production and ammonium, d) the ratio of catholyte ammonium to anolyte ammonium, e) pH changes.

To yield current from urine rich wastewater, acetate was added to the urine medium to act as an electron donor and the TKN was reduced to 4 g/L. During closed-circuit potential (CCP), the TKN traveled to the cathode. As shown in Figure 3a, two repetitions showed similar behavior but with different time intervals. Contribution of the organic nitrogen to the total nitrogen transfer was similar. Therefore, difference between repetition runs has originated from NH_4_-N fraction.

Power density curves for MEC reactors are shown in Figures 3b and 3c. The reactors began energy production at different times. As shown in Figure 3b, the power generation and urine hydrolysis started after 50 hours of operation. During this delay, nitrogen was transferred as organic form and diminished concentration gradient between anolyte and catholyte. A response in ammonium transmission to current was observed quickly after power production. NH_4_-N transport rates peaked when power increased, but this peak was also combined with ammonium peak in the anode. The ammonium transfer as a function of power density and ammonium content in the anode chamber are also presented in Figures 3b and 3c.

Before power generation, nitrogen had a chance to transfer as organic form. In the previous section, it was realized that the TKN transfer was independent of nitrogen type. Whatever the dominant nitrogen type was in the anode, the final TKN within cathode was similar in both series runs. As presented in Figures 3(a-c), during the current generation cycle, more than 45% of the all TKN transferred over a 150 h period occurred within 24 hours during which the current and urine dissociation were in maximum level. Urine dissociation into ammonium increased chance of nitrogen to pass through the membrane. Nitrogen should have been as ion form to take the responsibility of charge neutralization. Hence, as it was in organic form, the MEC could not have expedited its removal via transferring to the other chamber.

It was not very clear whether the transmission rate peak was due to current or ammonium concentration gradient due to coinciding of power production and urine dissociation peaks. To find whether the nitrogen equilibrium following current generation was a response to charge balance or it was a result of anolyte ammonium increase, the maximum ammonium concentrations in components for OCP and CCP were compared. Maximum ammonium concentration in the anode chamber with initial urine of 4 g-N/L reached 2680 mg-N/L; while, for this anolyte ammonium, 1580 mg-N/L ammonium was observed in the cathode (Figure 3d). In the second CCP run, anolyte and catholyte ammonium reached 2620 and 1460, respectively. Whereas upon OCP at 11 g-N/L initial urine, the ammonium in two different runs ranged from 2575 to 8345 mg-N/L in the anodes and 782 and 1260 mg-N/L in the cathodes, respectively. Although the initial concentrations of the ammonium in the CCP tests seemed to be lower than those shown by OCP tests, the transfer rates to the cathode were much higher. Based on anodic concentration, ammonium transferring upon CCP and OCP resulted in catholyte ammonium of 56-59% and 15-30% of anolyte ammonium, respectively. To understand the effect of current more clearly, the maximum catholyte ammonium increase rates occurred over 24-hour and 48-hour periods are provided in Table 2. Samples were taken every 24 hours.

pH was also changed during operations. pH increases in the anode compartment was deduced from the ammonium content and in the cathode from the ammonium transfer. pH changes in the cathode under CCP mode was mainly a result of current. Ammonium concentration in the anolyte always exceeded from the catolyte which raised pH only up to 9 (Figure 3e); However, catholyte pH in CCP increased up to 12. Following current drop, catholyte pH was decreased as much as 1–2 magnitudes.

Comparison between two modes disclosed the role of current on ammonium separation but dependency of ammonium upon ammonium dissociation made it difficult to distinguish the effect of current from diffusion and to define an absolute relationship for nitrogen transport through current within a urine rich wastewater. Controlling bacteria to hydrolyze urine in a constant or desirable rate is not easy. Hence, a series of short-term experiments were carried out with substrate containing ammonium (ammonium chloride) instead of urine. The results will be explained in the next section.

### Ammonium removal from ammonium rich substrate over OCP and CCP

To balance the charge in bioelectrochemical systems, as electrons transfer to the cathode, a movement of ions begins from or toward the anode based on the applied membrane. CEM is designed to transfer cation or to inhibit anion transportation. In this study, reactors used CEM; hence, protons or cations should have moved from the anode to the cathode.

**Figure 4:**
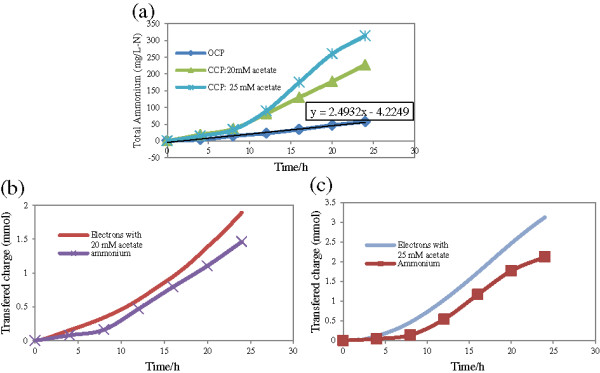
**Catholyte ammonium in two operation modes and its relationship with current; anodic medium contained 3 g-N/L NH**_**4**_**Cl: a) ammonium concentration, b & c) produced electrons and transferred ammoniums.**

Substrate containing 3000 mg-N/L was prepared to analyze ammonium transfer. Figure 4a shows ammonium transferring through CEM in different situations. OCP with no current had some ammonium transferred from the anode to the cathode. Total ammonium transfer over OCP resulted in 57 mg-N/L ammonium in the cathode within one day and a linear transfer over time was observed. Consequent equation stated that the cathode sucked 2.49 mg-N/L.h ammonium. Current speeded up the ammonium transfer, substantially. After one day, CCP showed 227 and 314 mg-N/L in the cathode based on two series runs, with similar ammonium content but different acetate content (20 and 25 mM). Over power production, ammonium removal curves were no longer linear. All ammonium transferred during CCP might not solely be due to charge neutralization and some of it might back to concentration gradient. OCP and CCP used the same medium. Assuming that the amount of ammonium transferred to the cathode resulting from diffusion is identical in both modes, the total ammonium transferred in OCP can be subtracted from the total ammonium transferred in CCP to find the fraction of ammonium attributed to the current. In mathematical words:

(4) $$NH_{4_{\mathrm{CCP}}}=NH_{4_{\mathrm{Current}}}+NH_{4_{\mathrm{Diffusion}}}$$

(5) DiffusionduringOCP=DifusionduringCCP

(6) $${\mathrm{NH}}_{4_{\mathrm{Current}}}={\mathrm{NH}}_{4_{\mathrm{CCP}}}‒{\mathrm{NH}}_{4_{\mathrm{OCP}}}$$

Considering that for each electron produced one proton should leave the anode, total ammonium was brought to mmol and was compared to cumulative current. The ammonium transport to the cathode during closed circuit potential is a function of current. Results can be seen in Figures 4b and 4c. Water loss and water intake during sampling were considered when calculating ammonium mass transfer. Over 24 hours, for 3.13 and 1.90 mmol electrons produced 2.12 and 1.46 mmol ammonium (29.7 and 20.5 mg-N) took the responsibility of charge neutralization.

As shown in Figures 4b and 4c, the share of current dedicated to ammonium removal decreased over time. This is perhaps due to proton concentration. In fact, the reason behind ion transferring instead of charge transferring is their higher concentration in the reactor other than protons [27]. In the current study, the reactors were operated in the batch modes; hence, anolyte ammonium decreased over time because of ammonium transmission to the cathode. Moreover, in the first day, current had increasing trend, which can be attributed to increase in proton concentration. Increase in the current and decrease in the ammonium concentration over time would diminish the ratio of ammonium to protons. Then, over time, protons were most probable to compete with ammonium and to slow down ammonium removal. Consequently, it can be concluded here that the fraction of current dedicated to ammonium separation decreased over time since the distance between ammonium removal curve and current production curve increased. This means α, the share of ammonium for charge balancing [*mmol NH*_4(*transferred*)_ = ∝ (*mmol electron*)_*produced*_], is a function of the current, ammonium concentration in the anode or even concentration of other ions. Comparison between Figures 4b and 4c supports this statement. These runs used the same ammonium concentration, but different acetate concentrations, lower acetate concentration produced lower current. Acetate was 20 mM in Figure 4b and 25 mM in Figure 4c; Clearly, the current curve and ammonium-transferred curve in Figure 4b are closer to each other than in Figure 4c. In both figures, the distance between current curve and ammonium curve increased over time. Current production rate was higher than ammonium removal rate. Although increase in the current decreased the contribution of the ammonium to charge neutralization, it improved the total ammonium removal as mg/L.

The nature of driving forces for ammonium transfer over OCP and CCP are different. Transfer of ammonium due to diffusion depends on concentration gradient between anolyte and catholyte. However, the ammonium transfer due to charge neutrality depends on the ratio of protons to the ammonium in the anode and is not related to ammonium concentration in the cathode. Hence, regardless of the limiting factors, physical ammonium removal is preferred for wastewaters with high ammonium concentration.

Ammonia stripping is a well-known process for ammonia removal from some industrial wastewaters over which pH is raised by addition of chemicals and the ammonium ion changes to ammonia and then air stripping brings out ammonia gas from wastewater. Reactions in MXC usually tend to increase pH in the cathode. The difference between catholyte pH in CCP mode and OCP mode was significant as shown in Figure 3e. Ammonium ion carries a hydrogen ion, although it is a basic compound and its transmission to the cathode causes more pH increase. In some cases, the cathodic pH did even reach 12. This pH enhances ammonium transformation to ammonia gas. Depending on temperature, more than 99% of the total ammonium is as ammonia form in pH 12 [28]. Blowing air in the air-cathode microbial fuel cells can also ease its removal afterward. Then ammonia stripping might happen in MFC.

## Conclusions

The role of current generation in bioelectrochemical systems on nitrogen fate and the potential of cation exchange membrane (CEM) for nitrogen transmission were addressed in this paper through an experimental work. Based on the results, urine and water could pass through CEM and transported between anode and cathode due to diffusion. The most portion of organic nitrogen transfer took place over the first two days. Ammonium was produced from urine and crossed the CEM; but, rates varied between OCP and CCP. Upon OCP while anolyte ammonium was 2575 and 8345 mg-N/L, catholyte ammonium was measured as 782 and 1260 mg-N/L. The catholyte NH_4_-N increased remarkably over current generation and terminated between 1580 and 1460 mg-N/L with the anolyte ammonium of 2680 and 2620 mg-N/L. Using ammonium chloride as a nitrogen source, CEM achieved a majority of charge balance by ammonium instead of protons and enabled ammonium removal. Difference between proton and ammonium concentrations was determining factor for ammonium separation. Of 3.13 and 1.90 mmol charges produced over a 24-hour period 2.12 and 1.46 mmol were balanced by ammonium. MEC systems made physical ammonium removal feasible during organic biodegradation and energy production. For a given reactor, if there is no limiting factor, physical ammonium removal by MEC systems would be better for higher nitrogen concentrations.

## Abbreviations

BES: Bioelectrochemical system; CCP: Closed-circuit potential; CEM: Cation exchange membrane; MEC: Microbial electrolysis cell; MFC: Microbial fuel cell; OCP: Open-circuit potential; TKN: Total Kjeldahl nitrogen.

## Competing interests

The authors declare that they have no competing interests.

## Authors’ contributions

SA carried out all experiments and tests. GN and NM were supervisors for experimental works. SA, GN and NM drafted the manuscript. All authors read and approved the final manuscript.
